# Association between Sjögren syndrome, sociodemographic factors, comorbid conditions, and optic neuritis: a Taiwanese population-based study

**DOI:** 10.3389/fneur.2024.1353326

**Published:** 2024-02-27

**Authors:** Ren-Long Jan, Chung-Han Ho, Cheng-Hao Sung, Jhi-Joung Wang, Han-Yi Jan, Wei-Yu Chen, Yuh-Shin Chang

**Affiliations:** ^1^Department of Medical Science Industries, College of Health Science, Chang Jung Christian University, Tainan, Taiwan; ^2^Department of Pediatrics, Chi Mei Medical Center, Liouying, Tainan, Taiwan; ^3^Department of Hospital and Health Care Administration, Chia Nan University of Pharmacy and Science, Tainan, Taiwan; ^4^Department of Medical Research, Chi Mei Medical Center, Tainan, Taiwan; ^5^Department of Ophthalmology, Chi Mei Medical Center, Tainan, Taiwan; ^6^College of Medicine, Tzu Chi University, Hualien, Taiwan; ^7^Department of Pediatrics, Chi Mei Medical Center, Tainan, Taiwan; ^8^College of Medicine, National Sun Yat-Sen University, Kaohsiung, Taiwan

**Keywords:** optic neuritis, Sjögren syndrome, case-controlled study, Taiwan longitudinal health insurance database, epidemiology

## Abstract

**Purpose:**

Our study aimed to explore the correlation between Sjögren syndrome, sociodemographic factors, comorbid conditions, and optic neuritis.

**Methods:**

This retrospective, nationwide, population-based, matched case–control investigation involved 33,190 individuals diagnosed with optic neuritis, identified using the International Classification of Diseases, Ninth Revision, Clinical Modification codes 377.30 for optic neuritis or 377.32 for retrobulbar neuritis. Patient data were extracted from the Taiwan National Health Insurance Research Database. Demographic characteristics, the presence of Sjögren syndrome, and pre-existing comorbid conditions were analyzed using univariate logistic regression. Continuous variables were assessed with a paired *t*-test. Adjusted logistic regression was employed to compare the prognosis odds ratio (OR) of patients with optic neuritis to controls.

**Results:**

After adjusting for confounding variables, individuals with Sjögren syndrome exhibited a significantly higher likelihood of developing optic neuritis compared to controls (adjusted OR, 9.79; 95% confidence interval [CI], 7.28–12.98; *p* < 0.0001). Other conditions associated with increased odds of optic neuritis included rheumatoid arthritis, ankylosing spondylitis, multiple sclerosis, systemic lupus erythematosus, and granulomatous vasculitis (adjusted OR: 1.57, 95% CI: 1.33–1.86; adjusted OR: 2.02, 95% CI: 1.65–2.48; adjusted OR: 140.77, 95% CI: 35.02–565.85; adjusted OR: 2.38, 95% CI: 1.71–3.30; adjusted OR: 18.28, 95% CI: 2.21–151.45, respectively), as well as systemic infections such as human herpes viral infection and tuberculosis infection (adjusted OR: 1.50, 95% CI: 1.35–1.66; adjusted OR: 4.60, 95% CI: 3.81–5.56, respectively).

**Discussion:**

Our findings strongly support the existence of an association between Sjögren syndrome, rheumatoid arthritis, ankylosing spondylitis, multiple sclerosis, systemic lupus erythematosus, granulomatous vasculitis, human herpes viral infection, tuberculosis, and optic neuritis.

## 1 Introduction

Optic neuritis, an inflammatory condition, demyelinates the optic nerve in one or both eyes and causes acute or subacute vision loss (1). Previously, our understanding of optic neuritis was based on the Optic Neuritis Treatment Trial (ONTT) and its results affected optic neuritis treatment worldwide (2, 3). Optic neuritis was categorized into typical type, i.e., idiopathic or multiple sclerosis related and with a good visual prognosis, and atypical type, i.e., not associated with multiple sclerosis and requiring corticosteroids for visual recovery. Recently, because of the discovery of the auto antibodies immunoglobulin G to aquaporin-4 and myelin oligodendrocyte glycoprotein, the importance of optic neuritis in neuromyelitis optica spectrum disorder and myelin oligodendrocyte glycoprotein antibody disease has become more prominent (1, 4, 5).

Although the pathophysiology of optic neuritis remains unclear, it is considered an immune-mediated disease (6) and could be a risk factor or the first clinical manifestation of autoimmune disease following systemic involvement (7). People may have a greater propensity for optic neuritis if they have underlying autoimmune diseases, including Sjögren syndrome (8, 9, 10), rheumatoid arthritis (8, 11), ankylosing spondylitis (7, 11, 12), multiple sclerosis (10, 13–15), systemic lupus erythematosus (7, 16, 17), and granulomatous vasculitis (10, 18–20). Moreover, some systemic infections, such as human herpes (21–24) and tuberculosis 10, 12, 25), with the ability to induce autoimmune reactions, may trigger optic neuritis attacks.

The cause and management of optic neuritis may vary according to the geographical location, ethnic background, and treatment availability, worldwide. Our aim was to elucidate the association between Sjögren syndrome, sociodemographic factors, various comorbid conditions (e.g., systemic infections, systemic autoimmune diseases, and optic neuritis) based on National Health Insurance database of Taiwan, containing records for >33,000 patients with optic neuritis.

## 2 Materials and methods

### 2.1 Database

Our cohort study utilized data from the National Health Insurance Research Database (NHIRD) in Taiwan, provided by the National Health Research Institutes (NHRI). The NHIRD includes encrypted patient identification numbers, demographic information (age, sex, residential area), and details on admission and discharge dates. It incorporates the International Classification of Diseases, Ninth Revision, Clinical Modification (ICD-9-CM) codes, covering procedures, diagnoses, prescription items, and associated costs funded by the NHRI. Approval exemption from the Institutional Review Board of the Chi Mei Medical Center was obtained.

### 2.2 Selection of patients and variables

**Figure 1:**
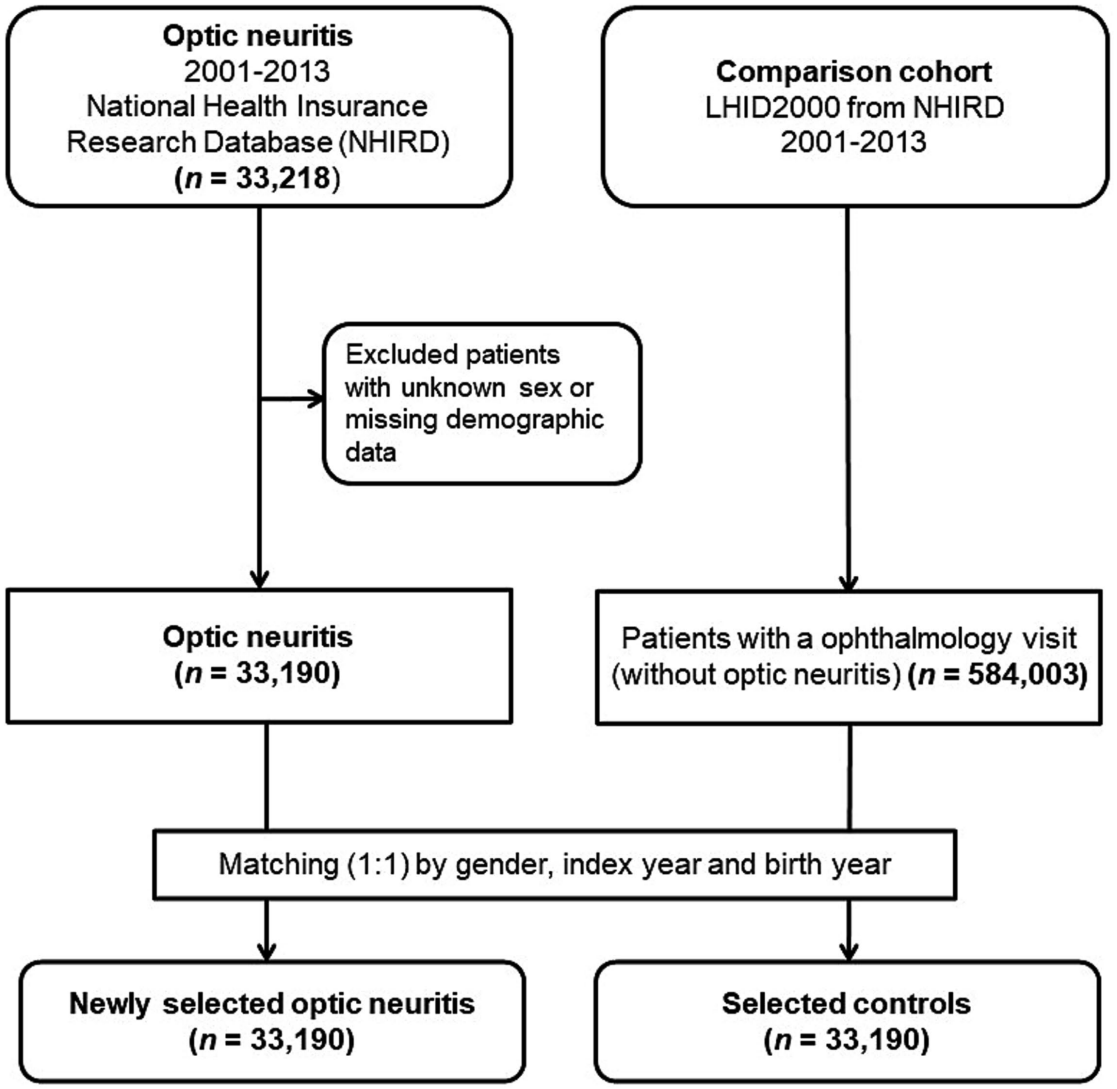
Flowchart outlining the enrollment process for patients with optic neuritis and the control patients.

This population-based case-controlled study enrolled a newly diagnosed optic neuritis group and a matched non-optic neuritis control group. Data were collected from January 1, 2001, to December 31, 2013. Figure 1 depicts the study’s flowchart. In the initial stage, 33,218 patients who received a diagnosis of optic neuritis (ICD-9-CM codes 377.30 for unspecified optic neuritis and 377.32 for retrobulbar neuritis) were considered for inclusion. After excluding individuals with unknown sex, missing demographic information, or those diagnosed with optic neuritis prior to January 1, 2001, a total of 33,190 patients were ultimately enrolled, and their data were extracted from the NHIRD.

For every individual diagnosed with optic neuritis, we randomly selected a control participant (an individual without optic neuritis) from the Longitudinal Health Insurance Database 2000. This specific database is a subset of the NHIRD, encompassing comprehensive claims data for one million beneficiaries throughout the year 2000. At the outset, we incorporated 584,003 individuals who had documented at least one visit to the ophthalmologist and lacked a prior diagnosis of optic neuritis before the index date from the pool of one million subjects in the LHID 2000, after excluding patients with missing sex or demographic data. The controls (*n* = 33,190) were matched to the optic neuritis patients based on age (±30 days), gender, and the index date, defined as the first day of the optic neuritis diagnosis. Controls were excluded if they had a diagnosis of optic neuritis before the specified index date. To assess the medical comorbidities associated with optic neuritis, we gathered data on various comorbid conditions, including systemic autoimmune diseases such as Sjögren’s syndrome (ICD-9-CM code 710.2), rheumatoid arthritis (ICD-9-CM code 714), ankylosing spondylitis (ICD-9-CM code 720), multiple sclerosis (ICD-9-CM code 340), systemic lupus erythematosus (ICD-9-CM codes 710.0 and 695.4), granulomatous vasculitis (sarcoidosis [ICD-9-CM code 135], and Wegener’s granulomatosis [ICD-9-CM code 446.4]), and systemic infectious conditions, including human herpes viral infection (varicella [ICD-9-CM codes 052.0–052.9], herpes zoster [ICD-9-CM codes 053–053.9], herpes simplex [ICD-9-CM codes 054–054.9 and 771.2], Epstein Barr virus [ICD-9-CM code 075], and cytomegaloviral disease [ICD-9-CM code 078.5]). Additionally, tuberculosis (ICD-9-CM code 010–018) and syphilis (ICD-9-CM codes 090–097) were included. Identification of these comorbidities relied on the presence of an ICD-9-CM code recorded within 1 year before the index date, confirmed through three or more ambulatory care claims or inpatient admittance.

### 2.3 Statistical analysis

All statistical analyses were conducted utilizing SAS 9.4 for Windows, developed by SAS Institute, Inc., located in Cary, NC, United States. Demographic characteristics such as age group, sex, income, geographic region, residential city status, and occupation were analyzed using McNemar’s test. For continuous variables, a paired *t*-test was employed. Furthermore, the comparison of comorbid conditions between patients with optic neuritis and controls utilized McNemar’s test. Odds ratios (ORs) were computed through univariate logistic regression analyses, and a multivariable logistic regression model, based on age, sex, and the index date, was employed to calculate adjusted ORs for various comorbidities associated with a diagnosis of optic neuritis. The independent variables considered in the analyses encompassed sociodemographic factors (income, geographic region, residential city status, and occupation) and all aforementioned medical conditions of interest. The level of significance for all analyses was set at *p* < 0.05.

## 3 Results

### 3.1 Demographic data

#### 3.1.1 Sociodemographic factors

After excluding ineligible patients, our analysis focused on 33,190 individuals diagnosed with optic neuritis, alongside an equal number of age and sex-matched controls who utilized medical care services covered by the NHRI from 2001 to 2013. The mean age of both patients with optic neuritis and controls was 50.13 years, with a standard deviation of 15.75 (see Table 1). Within the cohort of 33,190 optic neuritis patients, 14,143 (42.61%) were male, while 19,047 (57.39%) were female.

**Table 1:** Baseline socio-demographic factors and comorbid conditions of optic neuritis patients and age-and sex-matched control participants.

	Optic neuritis *N* = 33,190	Comparison *N* = 33,190	*p*-value
**Socio-demographic factors**	*n* (%)	*n* (%)	
Age (years; mean ± SD)	50.13 ± 15.75	50.13 ± 15.75	1.0000^a^
Age (years)			
<25	1,801 (5.43)	1,801 (5.43)	1.0000^b^
25–34	4,105 (12.37)	4,105 (12.37)	
35–44	5,945 (17.91)	5,945 (17.91)	
45–54	8,118 (24.46)	8,118 (24.46)	
55–64	6,825 (20.56)	6,825 (20.56)	
≥65	6,396 (19.27)	6,396 (19.27)	
Sex			
Male	14,143 (42.61)	14,143 (42.61)	1.0000^b^
Female	19,047 (57.39)	19,047 (57.39)	
Income			<0.0001^b^
<NT$ 30,000	19,573 (58.97)	20,031 (60.35)	
NT$ 30,000–60,000	11,073 (33.36)	10,567 (31.84)	
NT$ 60,000–90,000	2,009 (6.05)	1,971 (5.94)	
NT$ 90,000–120,000	280 (0.84)	337 (1.02)	
>NT$ 120,000	255 (0.77)	284 (0.86)	
Geographical region of Taiwan			<0.0001^b^
Northern	14,762 (44.48)	16,699 (50.31)	
Central	6,492 (19.56)	6,343 (19.11)	
Southern	11,169 (33.65)	9,239 (27.84)	
Eastern	767 (2.31)	909 (2.74)	
Residential city status			<0.0001^b^
Metropolis	24,024 (72.38)	23,592 (71.08)	
Satellite	2,718 (8.19)	2,256 (6.80)	
Rural	6,448 (19.43)	7,342 (22.12)	
Occupation			<0.0001^b^
Public servant	17,157 (51.69)	17,418 (52.48)	
Farmer	3,930 (11.84)	4,320 (13.02)	
Fisherman	841 (2.53)	663 (2.00)	
Others	11,262 (33.93)	10,789 (32.51)	
**Comorbid conditions**			
Autoimmune disease			
Sjögren syndrome	593 (1.79)	55 (0.17)	<0.0001^b^
Rheumatoid arthritis	445 (1.34)	234 (0.71)	<0.0001^b^
Ankylosing spondylitis	313 (0.94)	142 (0.43)	<0.0001^b^
Multiple sclerosis	299 (0.90)	2 (0.01)	<0.0001^b^
Systemic lupus erythematosus	173 (0.52)	51 (0.15)	<0.0001^b^
Granulomatous vasculitis	18 (0.05)	1 (0.00)	<0.0001^b^
Systemic infection			
Herpes infection	987 (2.97)	619 (1.87)	<0.0001^b^
Tuberculosis	636 (1.92)	138 (0.42)	<0.0001^b^
Syphilis	40 (0.12)	26 (0.08)	0.0847

a Paired *t*-test.

b McNemar’s test.

NT$, New Taiwan dollars; SD, standard deviation.

Significant differences emerged in the income distribution between patients with optic neuritis and controls (*p* < 0.0001). The predominant income bracket for patients with optic neuritis was below 30,000 New Taiwan dollars (NT$), constituting 58.97% of the cohort (19,573 individuals). Geographic distribution also displayed a notable dissimilarity between the two groups (*p* < 0.0001). Northern Taiwan emerged as the most common region of residence for those diagnosed with optic neuritis, accounting for 44.48% of cases (*n* = 14,762). Examining urban–rural disparities, a substantial majority of patients with optic neuritis resided in metropolis cities (*n* = 24,024; 72.38%), a statistically significant difference when compared to those in rural areas (*n* = 6,448; 19.43%) and satellite cities (*n* = 2,718; 8.19%). Occupational classification further underscored distinctions in patients with optic neuritis. Notably, more than half of the 33,190 patients with optic neuritis held positions as public servants, including military, civil, or teaching staff (*n* = 17,157; 51.69%).

#### 3.1.2 Comorbid conditions

The patients with optic neuritis exhibited a significantly higher prevalence of autoimmune diseases, such as Sjögren syndrome (*n* = 593; 1.79%; *p* < 0.0001), rheumatoid arthritis (*n* = 445; 1.34%; *p* < 0.0001), ankylosing spondylitis (*n* = 313; 0.94%; *p* < 0.0001), multiple sclerosis (*n* = 299; 0.90%; *p* < 0.0001), systemic lupus erythematosus (*n* = 173; 0.52%; *p* < 0.0001), and granulomatous vasculitis (*n* = 18; 0.05%; *p* < 0.0001) between the patients with optic neuritis and the controls. There was a significantly higher prevalence of systemic infections in patients with optic neuritis, including human herpes viral infections (*n* = 987; 2.97%; *p* < 0.0001), tuberculosis (*n* = 636; 1.92%; *p* < 0.0001), and syphilis (*n* = 40; 0.12%; *p* < 0.0001) compared to that in controls (Table 1).

### 3.2 Associated risk factors

#### 3.2.1 Sociodemographic factors

We employed univariate logistic regression analyses and a multiple logistic regression model, adjusting for age, sex, sociodemographic factors, and comorbidities, to examine the sociodemographic factors—specifically income, geographic region, residential city status, and occupation—of patients with optic neuritis and controls. The results are presented in Table 2.

**Table 2:** Odds ratios and adjusted odds ratios of various socio-demographic factors and comorbid conditions for optic neuritis.

	Odds ratio^a^ (95% CI)	*p*-value	Adjusted odds ratio^b^ (95% CI)	*p*-value
**Socio-demographic factors**				
Income				
<NT$ 30,000	1.00		1.00	
NT$ 30,000–60,000	1.08 (1.04–1.12)	<0.0001	1.14 (1.10–1.19)	<0.0001
NT$ 60,000–90,000	1.05 (0.98–1.13)	0.1358	1.14 (1.06–1.22)	0.0004
NT$ 90,000–120,000	0.86 (0.74–1.01)	0.0695	0.96 (0.82–1.14)	0.6644
>NT$ 120,000	0.93 (0.78–1.10)	0.4006	1.07 (0.90–1.28)	0.4294
Geographical region of Taiwan				
Northern	1.05 (0.95–1.15)	0.3875	0.90 (0.81–1.00)	0.0499
Central	1.22 (1.10–1.35)	0.0002	1.13 (1.01–1.26)	0.0299
Southern	1.43 (1.30–1.58)	<0.0001	1.28 (1.15–1.43)	<0.0001
Eastern	1.00		1.00	
Residential city status				
Metropolis	1.00		1.00	
Satellite	1.18 (1.12–1.25)	<0.0001	1.29 (1.21–1.37)	<0.0001
Rural	0.86 (0.83–0.89)	<0.0001	0.76 (0.73–0.79)	<0.0001
Occupation				
Public servant	0.94 (0.91–0.98)	0.0014	0.93 (0.89–0.96)	0.0001
Farmer	0.86 (0.81–0.91)	<0.0001	0.84 (0.80–0.89)	<0.0001
Fisherman	1.22 (1.10–1.35)	0.0003	1.16 (1.04–1.30)	0.0063
Others	1.00		1.00	
**Comorbid conditions**				
Autoimmune disease				
Sjögren syndrome	10.96 (8.30–14.49)	<0.0001	9.79 (7.38–12.98)	<0.0001
Rheumatoid arthritis	1.92 (1.64–2.25)	<0.0001	1.57 (1.33–1.86)	<0.0001
Ankylosing spondylitis	2.21 (1.81–2.70)	<0.0001	2.02 (1.65–2.48)	<0.0001
Multiple sclerosis	149.50 (32.22–600.54)	<0.0001	140.77 (35.02–565.85)	<0.0001
Systemic lupus erythematosus	3.39 (2.48–4.64)	<0.0001	2.38 (1.71–3.30)	<0.0001
Granulomatous vasculitis	18.00 (2.40–134.83)	0.0049	18.28 (2.21–151.45)	0.0071
Systemic infection				
Herpes infection	1.61 (1.45–1.78)	<0.0001	1.50 (1.35–1.66)	<0.0001
Tuberculosis	4.72 (3.91–5.68)	<0.0001	4.60 (3.81–5.56)	<0.0001
Syphilis	1.54 (0.94–2.52)	0.0873	1.51 (0.92–2.49)	0.1068

a Odds ratios were obtained from univariate logistic regression analyses.

b Adjusted odds ratio were calculated from a multivariable logistic regression model that was conditioned on age-group, sex, and the year of index date.

CI, confidence interval; NT$, New Taiwan dollars.

Patients with monthly incomes ranging from NT$ 30,000–60,000 and 60,000–90,000 exhibited increased odds of developing optic neuritis compared to those with an income <NT$ 30,000, even after adjusting for other confounding factors. Concerning geographic location, patients residing in Central or Southern Taiwan demonstrated a significantly higher prevalence of optic neuritis compared to those in Eastern Taiwan. This geographic difference remained a significant risk factor following a conditional logistic regression analysis. Examining residential city status, patients living in a satellite city exhibited a significantly higher prevalence of optic neuritis relative to those in a metropolis city, even after conducting a conditional logistic regression analysis. Occupationally, individuals engaged in fishing faced a significant risk of developing optic neuritis, an independent risk factor even after considering other confounders, as indicated in Table 2.

#### 3.2.2 Comorbid conditions

We conducted both univariate and multiple logistic regression analyses to explore several potential comorbidities, as detailed in Table 2. Patients with autoimmune diseases, such as Sjögren syndrome, rheumatoid arthritis, ankylosing spondylitis, multiple scleritis, systemic lupus erythematosus, and granulomatous vasculitis, exhibited significantly higher odds ratios (ORs) for receiving an optic neuritis diagnosis (OR: 10.96, 95% CI: 8.30–14.49, *p* < 0.0001; OR: 1.92, 95% CI: 1.64–2.25, *p* < 0.0001; OR: 2.21, 95% CI: 1.81–2.70, *p* < 0.0001; OR: 149.50, 95% CI: 32.22–600.54, *p* < 0.0001; OR: 3.39, 95% CI: 2.48–4.64, *p* < 0.0001; OR: 18.00, 95% CI: 2.40–134.83, *p* = 0.0049, respectively). These associations remained significant even after conducting conditional logistic regression (adjusted OR: 9.79, 95% CI: 7.38–12.98, *p* < 0.0001; adjusted OR: 1.57, 95% CI: 1.33–1.86, *p* < 0.0001; adjusted OR: 2.02, 95% CI: 1.65–2.48, *p* < 0.0001; adjusted OR: 140.77, 95% CI: 35.02–565.85, *p* < 0.0001; adjusted OR: 2.38, 95% CI: 1.71–3.30, *p* < 0.0001; adjusted OR: 18.28, 95% CI: 2.21–151.45, *p* = 0.0071, respectively).

Patients with systemic infections, including human herpes viral infection and tuberculosis, also had significantly increased odds of an optic neuritis diagnosis both before (OR: 1.61, 95% CI: 1.45–1.78, *p* < 0.0001; OR: 4.72, 95% CI: 3.91–5.68, *p* < 0.0001, respectively) and after adjustment for other confounders (adjusted OR: 1.50, 95% CI: 1.35–1.66, *p* < 0.0001; adjusted OR: 4.60, 95% CI: 3.81–5.56, *p* < 0.0001, respectively).

**Figure 2:**
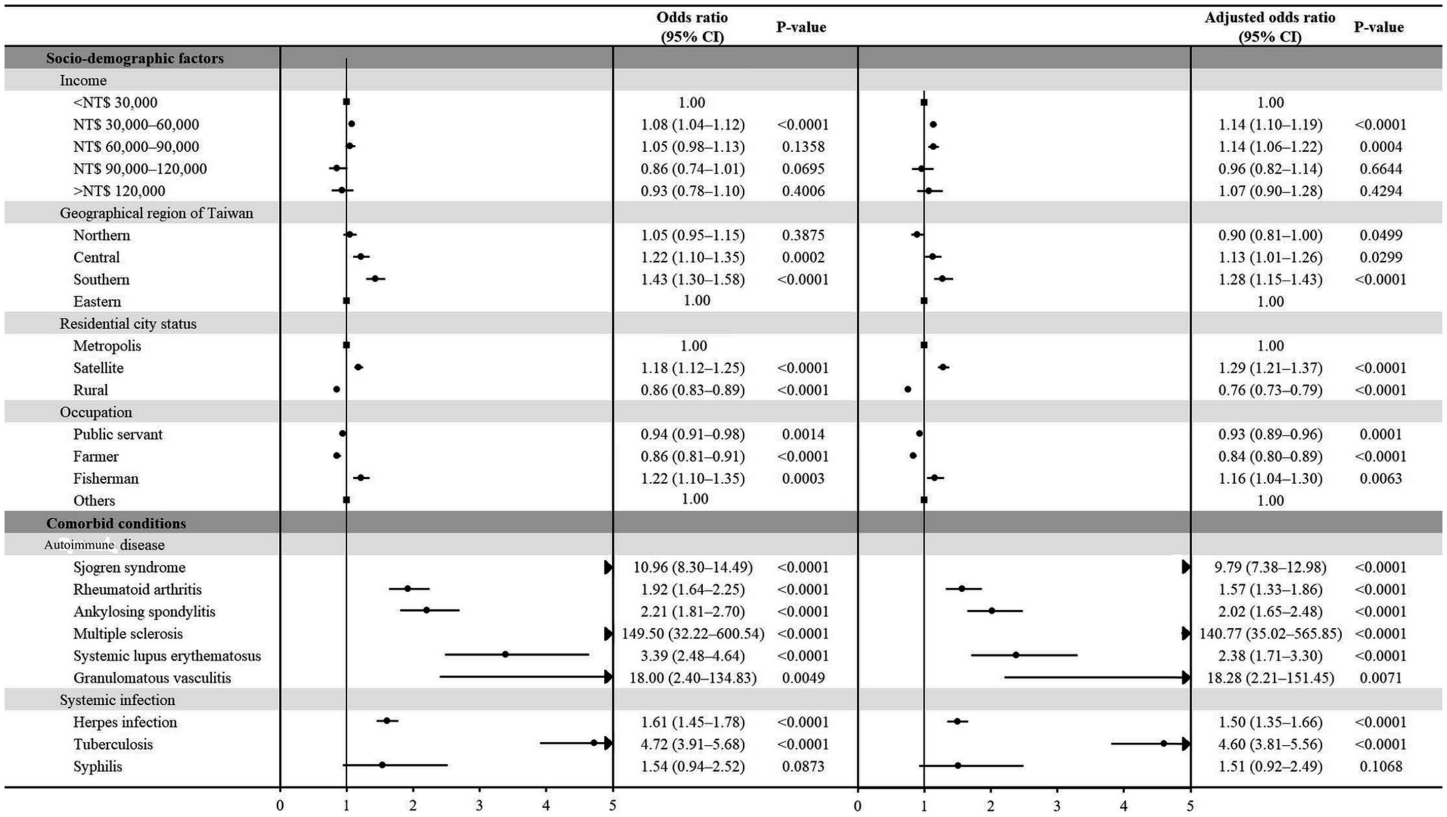
Forest plot depicting the odds ratios (ORs) and adjusted ORs related to diverse socio-demographic factors and comorbid conditions linked with optic neuritis.

We have added the forest plot for Table 2 to illustrate the ORs and adjusted ORs of various socio-demographic factors and comorbid conditions associated with optic neuritis (Figure 2).

## 4 Discussion

To the best of our knowledge, this study stands as the most extensive nationwide, population-based, case–controlled investigation assessing the relationship between sociodemographic factors, prevalent comorbid conditions, and optic neuritis. Our analyses found that optic neuritis was more common in female individuals, with a 57.39% predominance. In addition, some comorbid conditions significantly influenced the odds of developing optic neuritis. In comparison, patients who had a systemic autoimmune disease, or were in a systemic infectious state had significantly higher odds of developing optic neuritis. Interestingly, patients with Sjögren syndrome had considerably higher odds of developing optic neuritis compared to those who did not have the syndrome (adjusted OR: 9.79, 95% CI: 7.38–12.98, *p* < 0.0001).

Of the 33,190 patients with optic neuritis, the average age at optic neuritis diagnosis was 50.13 years (SD: 15.75), in accordance with a previous Taiwanese retrospective cohort study (7). However, this finding was inconsistent with the ONTT, which reported that optic neuritis affects patients at a young age (mean age: 31.8 years) (26, 27). The exclusion of participants with bilateral optic neuritis in the ONTT may play a role in the aforementioned inconsistency. The variation in age observed between our findings and previous reports could be attributed to several potential factors. Racial diversity, variances in geographic distribution, environmental influences, and disparities in medical insurance systems—including differences in medical information, resources, and accessibility—may collectively contribute to the differences in the age distribution of optic neuritis diagnoses.

The patients with optic neuritis demonstrated a female preponderance (57.39%) in the current study, which was consistent with previously reported female predominance (10, 26–28). We speculate that the reason of higher prevalence of optic neuritis in women may be related to sex-dependent susceptibility to autoimmune diseases, which is an important etiology of optic neuritis development (7, 10). Whether sex hormones are involved in the pathophysiology of optic neuritis requires further investigation.

Upon considering sociodemographic factors beyond age and sex, we observed a notably lower OR for optic neuritis among individuals residing in Eastern Taiwan compared to those in Southern and Central Taiwan, particularly in rural areas when contrasted with metropolis cities. The reduced incidence of optic neuritis diagnoses in Eastern Taiwan and rural settings within our study may reflect challenges such as limited access to medical care, reluctance to seek ophthalmological consultations, and a shortage of neuro-ophthalmology specialists for the diagnosis and management of optic neuritis. These challenges may be more pronounced in comparison to other regions in Taiwan and different residential city statuses. Additionally, individuals with an income range of NT$30,000–90,000 exhibited significantly higher odds of developing optic neuritis. The optic neuritis activity or risk is affected by various socioeconomic factors, including income, educational level, and living conditions. Individuals with higher income levels may possess greater knowledge and awareness of optic nerve disorders, leading them to promptly and proactively seek assistance when encountering ophthalmic issues. Consequently, socioeconomic status may impact the timely initiation of therapy and follow-up for patients managing optic neuritis.

In the present study, patients diagnosed with Sjögren syndrome exhibited a notably elevated OR for the development of optic neuritis (adjusted OR: 7.97, 95% CI: 7.38–12.98, *p* < 0.0001). Sjögren syndrome, a prevalent autoimmune disease, is characterized by chronic inflammation mediated through autoantibody production and lymphocytic infiltration (29). This immune-mediated systemic inflammatory condition is characterized by autoimmune exocrinopathy, mainly affecting the salivary and lacrimal glands leading to xerostomia and xerophthalmia (29). Our study identified Sjögren syndrome as a prominent independent risk factor for the formation of optic neuritis. This association has been previously reported in several studies (8, 9, 10). Akpek et al. conducted a longitudinal cohort study including 163 patients with definitively diagnosed primary Sjögren syndrome and reported that three patients developed optic neuritis (2%) (9). Another retrospective matched cohort study, which was conducted by Tasanee et al., included 2,893 patients with optic neuritis. The authors reported that patients with optic neuritis had a significantly higher hazard of incident Sjögren syndrome (hazard ratio [HR]: 3.48, 95% CI: 1.38–8.76) (10). Interestingly, optic neuritis may present even in the absence of typical sicca symptoms initially in patients with Sjögren syndrome (30). Nitescu et al. advocated the routine screening of anti SSA and SSB in patients with optic neuritis (31). A timely referral for systemic workups with a close collaboration between ophthalmologists and rheumatologists is important to reduce delays in the diagnosis and improve the quality of life of patients with Sjögren syndrome.

Although rheumatoid arthritis following optic neuritis did not reach statistical significance (adjusted HR: 1.06, 95% CI: 0.66–1.69) in a previous cohort study (7), we found that rheumatoid arthritis plays a role in optic neuritis development. Patients with rheumatoid arthritis had a significantly higher OR for optic neuritis development (adjusted OR: 1.57, 95% CI: 1.33–1.86, *p* < 0.0001). An association between patients with rheumatoid arthritis and optic neuritis has been sparsely reported previously. Yokoyama et al. presented a case with an acute onset of optic neuritis after a long period of etanercept use for rheumatoid arthritis, and reported that demyelination could develop long after anti-tumor necrosis factor agents (11). Li et al. conducted a clinical study to assess frequency of autoantibodies of connective tissue diseases in patients with optic neuritis, and found that recurrent optic neuritis is often associated with rheumatoid factor, a serological marker of rheumatoid arthritis (8).

With respect to other immune-mediated systemic inflammatory conditions, we found that patients with ankylosing spondylitis had a significantly higher OR for optic neuritis development (adjusted OR: 2.02, 95% CI: 1.65–2.48, *p* < 0.0001) (7, 11, 12). Ankylosing spondylitis, a chronic inflammatory rheumatic disorder, is well known in relation to acute anterior uveitis which is the most common ophthalmologic involvement of ankylosing spondylitis. The association between optic neuritis and ankylosing spondylitis is rarely mentioned and reported by some reports only (7, 11, 12). Ma et al. analyzed 1,847 patients with optic neuritis and 7,388 controls and found that ankylosing spondylitis was an independent risk factor of optic neuritis (adjusted HR: 2.86, 95% CI: 1.54–5.31) (7). Optic neuritis may be initial presentation of ankylosing spondylitis (32), or concurrent with active ankylosing spondylitis (33, 34). These studies hypothesized the association based on the underlying link between HLA-B27 and optic neuritis.

We found that, compared to the control group, patients with multiple sclerosis had a significantly higher risk of developing optic neuritis (adjusted OR: 140.77, 95% CI: 35.02–565.85, *p* < 0.0001). This finding concurred with the results of previous studies (10, 13–15). Tasanee et al., reported that patients with optic neuritis had significantly higher odds of prior multiple sclerosis (OR: 98.22, 95% CI: 65.40–147.52) in their case–control study, and had a significantly higher hazard of multiple sclerosis (HR: 284.97, 95% CI: 167.85–483.81) in their cohort study (10). Multiple sclerosis, a central nervous system neurodegenerative and demyelinating disease, is influenced by autoimmune, genetic or environmental factors, and ocular involvement is ubiquitously marked by the presence of optic neuritis (35–37). Optic neuritis is frequent in the evolution of multiple sclerosis as up to 70% of patients with multiple sclerosis reportedly have an acute episode of optic neuritis during their course (35). The association is based on various pathophysiological mechanisms, such as demyelination, inflammation, or axonal degeneration (13, 35). It is important to diagnose optic neuritis in multiple sclerosis so that treatment can be started promptly and possibly reduce the visual impairment.

In accordance with previous reports, systemic lupus erythematosus is another significant risk factor for optic neuritis (adjusted OR: 2.38, 95% CI: 1.71–3.30, *p* < 0.0001) (7, 16). Systemic lupus erythematosus is a chronic multisystem autoimmune disease and as many as one-third of systemic lupus erythematosus patients experience ophthalmic symptoms. Optic neuritis is a relatively rare manifestation of systemic lupus erythematosus, and is caused by an ischemic process that may result in subsequent demyelination (17). Lin et al. found that systemic lupus erythematosus-related optic neuritis often has severe visual impairment and needs early administration of corticosteroids (16). Ophthalmologists who care for patients suffering from systemic lupus erythematosus should differentiate systemic lupus erythematosus-related optic neuritis from idiopathic optic neuritis, as early diagnosis and prompt treatment are crucial for restoring visual function in these patients (16).

Granulomatous vasculitis including sarcoidosis and Wegener’s granulomatosis was also found to be an independent risk factor for optic neuritis after adjusting for other confounders (adjusted OR: 18.28, 95% CI: 2.21–151.45, *p* = 0.0071). This finding is consistent with results of several previous studies which reports the association between optic neuritis and Wegener’s granulomatosis (10, 18–20), or sarcoidosis (38, 39). Sarcoidosis is a chronic multisystemic inflammatory disease that commonly affects the visual and neurological systems: the most common neuro-ophthalmic manifestation is optic neuropathy. Optic neuritis has been considered as the most common manifestation of neuro-sarcoidosis ranging from 30 to 70% in a previous report (40). Sarcoid-related optic neuritis is an important differential diagnosis in typical demyelinating optic neuritis associated with multiple sclerosis and atypical optic neuritis, especially in the younger age group (39).

In the current study, approximately 5% of optic neuritis diagnoses were associated with systemic infectious state, such as human herpes virus infection and tuberculosis. Patients with human herpes virus infection (adjusted OR: 1.50, 95% CI: 1.35–1.66, *p* < 0.0001) and tuberculosis (adjusted OR: 4.60, 95% CI: 3.81–5.56, *p* < 0.0001) had a significantly higher OR for optic neuritis development. A recent human herpes viral infection may increase the risk of optic neuritis formation. Our finding is in accordance with several reports, whose results point out the potential role of human herpes viruses infection in optic neuritis and suggest the various herpes viruses as triggering agents of autoimmunity (21–24). In addition to human herpes virus infection, tuberculosis also plays a role in optic neuritis development. An association between patients with optic neuritis and tuberculosis has been reported by several studies and suggested that pulmonary tuberculosis-related neuromyelitis optica is caused by an immune response to tuberculosis infection (10, 12, 25).

Our study had several strengths. Firstly, it stands as the largest investigation to date, focusing on the association between Sjögren syndrome and optic neuritis. The risk of selection bias from referral centers was mitigated, given that the data were sourced from a nationwide and population-based dataset. Moreover, the study eliminated recall bias by relying on electronically recorded data in the NHIRD database rather than patient self-reporting. Utilizing 13 years of longitudinal data, the case-control study thoroughly examined various sociodemographic factors, systemic autoimmune diseases, and systemic infectious states in both patients with optic neuritis and controls. The study ensured the reliability of results by appropriately adjusting for confounding factors when calculating odds ratios (ORs) in patients with optic neuritis.

However, certain limitations should be acknowledged. Firstly, optic neuritis and comorbid disorders were diagnosed solely based on ICD-9-CM codes, potentially leading to disease misclassification. Furthermore, the study could not confirm the presence of optic neuritis in the patient group or its absence in the control group due to the lack of access to clinical records. Future research should address these limitations by incorporating sociodemographic and pathophysiologic factors, clinical information, and questionnaires. Lastly, the medical history could only be traced back to 1996, making it impossible to confirm whether controls had been diagnosed with optic neuritis before January 1996.

In summary, our study revealed that residing in Eastern Taiwan and rural areas was associated with a reduced risk of optic neuritis. Interestingly, patients with optic neuritis in Taiwan did not seem to face employment or income limitations due to the disease. After accounting for sociodemographic factors and potential comorbidities, our findings highlighted significantly higher risks of developing optic neuritis in patients with Sjögren syndrome, rheumatoid arthritis, ankylosing spondylitis, multiple sclerosis, systemic lupus erythematosus, granulomatous vasculitis, human herpes viral infection, and tuberculosis.

## Data availability statement

The original contributions presented in the study are included in the article/supplementary material, further inquiries can be directed to the corresponding author.

## Ethics statement

The studies involving humans were approved by Institutional Review Board of the Chi Mei Medical Center. The studies were conducted in accordance with the local legislation and institutional requirements. Written informed consent for participation was not required from the participants or the participants’ legal guardians/next of kin in accordance with the national legislation and institutional requirements.

## Author contributions

R-LJ: Conceptualization, Formal analysis, Methodology, Writing – original draft, Writing – review & editing. C-HH: Formal analysis, Methodology, Writing – original draft, Writing – review & editing. C-HS: Writing – original draft, Writing – review & editing. J-JW: Software, Writing – original draft, Writing – review & editing, Resources. H-YJ: Writing – original draft, Writing – review & editing. W-YC: Writing – original draft, Writing – review & editing. Y-SC: Conceptualization, Formal analysis, Methodology, Writing – original draft, Writing – review & editing.

## References

[1] Beck RW Cleary PA Anderson MM Jr Keltner JL Shults WT Kaufman DI. A randomized, controlled trial of corticosteroids in the treatment of acute optic neuritis. The optic neuritis study group. N Engl J Med. (1992) 326:581–8. doi: 10.1056/NEJM199202273260901 1734247

[2] Benard-Seguin E Costello F. Optic neuritis: current challenges in diagnosis and management. Curr Opin Neurol. (2023) 36:10–8. doi: 10.1097/WCO.0000000000001128, PMID: 36630210

[3] Hickman SJ Petzold A. Update on optic neuritis: an international view. Neuroophthalmology. (2022) 46:1–18. doi: 10.1080/01658107.2021.1964541, PMID: 35095131 PMC8794242

[4] Lennon VA Wingerchuk DM Kryzer TJ Pittock SJ Lucchinetti CF Fujihara K. A serum autoantibody marker of neuromyelitis optica: distinction from multiple sclerosis. Lancet. (2004) 364:2106–12. doi: 10.1016/S0140-6736(04)17551-X, PMID: 15589308

[5] Wingerchuk DM Banwell B Bennett JL Cabre P Carroll W Chitnis T. International consensus diagnostic criteria for neuromyelitis optica spectrum disorders. Neurology. (2015) 85:177–89. doi: 10.1212/WNL.0000000000001729, PMID: 26092914 PMC4515040

[6] Roed H Frederiksen J Langkilde A Sorensen TL Lauritzen M Sellebjerg F. Systemic T-cell activation in acute clinically isolated optic neuritis. J Neuroimmunol. (2005) 162:165–72. doi: 10.1016/j.jneuroim.2005.02.002, PMID: 15833372

[7] Ma KS Lee CM Chen PH Yang Y Dong YW Wang YH. Risk of autoimmune diseases following optic neuritis: a Nationwide population-based cohort study. Front Med (Lausanne). (2022) 9:903608. doi: 10.3389/fmed.2022.903608, PMID: 35770018 PMC9234206

[8] Li H Zhang Y Yi Z Huang D Wei S. Frequency of autoantibodies and connective tissue diseases in Chinese patients with optic neuritis. PLoS One. (2014a) 9:e99323. doi: 10.1371/journal.pone.0099323, PMID: 24950188 PMC4064964

[9] Akpek EK Mathews P Hahn S Hessen M Kim J Grader-Beck T. Ocular and systemic morbidity in a longitudinal cohort of Sjögren's syndrome. Ophthalmology. (2015) 122:56–61. doi: 10.1016/j.ophtha.2014.07.026, PMID: 25178806

[10] Braithwaite T Subramanian A Petzold A Galloway J Adderley NJ Mollan SP. Trends in optic neuritis incidence and prevalence in the UK and association with systemic and neurologic disease. JAMA Neurol. (2020) 77:1514–23. doi: 10.1001/jamaneurol.2020.3502, PMID: 33017023 PMC7536630

[11] Yokoyama W Takada K Miyasaka N Kohsaka H. Myelitis and optic neuritis induced by a long course of etanercept in a patient with rheumatoid arthritis. BMJ Case Rep. (2014) 2014:bcr2014205779. doi: 10.1136/bcr-2014-205779, PMID: 25085953 PMC4127673

[12] Zhao S Zhou H Peng X Zhu J Wang W Kang H. Optic neuritis with positive HLA-B27: characteristic phenotype in the Chinese population. J Neurol Sci. (2016) 362:100–5. doi: 10.1016/j.jns.2016.01.027, PMID: 26944126

[13] Ciapa MA Salaru DL Statescu C Sascau RA Bogdanici CM. Optic neuritis in multiple sclerosis-a review of molecular mechanisms involved in the degenerative process. Curr Issues Mol Biol. (2022) 44:3959–79. doi: 10.3390/cimb44090272, PMID: 36135184 PMC9497878

[14] Lin WS Chen HM Yang CC Chen TC Lin JW Lee WT. Multiple sclerosis and neuromyelitis optica after optic neuritis: a nationwide cohort study in Taiwan. Mult Scler Relat Disord. (2020) 44:102379. doi: 10.1016/j.msard.2020.102379, PMID: 32650124

[15] Osborne BJ Volpe NJ. Optic neuritis and risk of MS: differential diagnosis and management. Cleve Clin J Med. (2009) 76:181–90. doi: 10.3949/ccjm.76a.07268, PMID: 19258465

[16] Lin YC Wang AG Yen MY. Systemic lupus erythematosus-associated optic neuritis: clinical experience and literature review. Acta Ophthalmol. (2009) 87:204–10. doi: 10.1111/j.1755-3768.2008.01193.x, PMID: 18507726

[17] Lubon W Lubon M Kotyla P Mrukwa-Kominek E. Understanding ocular findings and manifestations of systemic lupus erythematosus: update review of the literature. Int J Mol Sci. (2022) 23:12264. doi: 10.3390/ijms232012264, PMID: 36293119 PMC9603180

[18] Huchzermeyer C Mardin C Holbach L Zwerina J Schett G Rech J. Successful remission induction with a combination therapy of rituximab, cyclophosphamide, and steroids in a patient with refractory optic neuritis in Wegener's granulomatosis. Clin Rheumatol. (2013) 32:97–101. doi: 10.1007/s10067-010-1561-9 20862503

[19] Moubayed SP Black DO. Optic neuritis as an initial presentation of Wegener's granulomatosis. Can J Ophthalmol. (2009) 44:e59. doi: 10.3129/i09-145, PMID: 20051998

[20] Niskopoulou M Du Toit N. Optic neuritis as a feature of Wegener's granulomatosis. Eye (Lond). (2002) 16:320–1. doi: 10.1038/sj.eye.6700096, PMID: 12032725

[21] Goswami M Bhattacharya S Bandyopadhyay M. Ocular manifestation and visual outcomes in herpes zoster ophthalmicus: a prospective study from a tertiary hospital of eastern India. Int J Ophthalmol. (2021) 14:1950–6. doi: 10.18240/ijo.2021.12.21, PMID: 34926213 PMC8640756

[22] Kahloun R Attia S Jelliti B Attia AZ Khochtali S Yahia SB. Ocular involvement and visual outcome of herpes zoster ophthalmicus: review of 45 patients from Tunisia, North Africa. J Ophthalmic Inflamm Infect. (2014) 4:25. doi: 10.1186/s12348-014-0025-9, PMID: 25246984 PMC4169054

[23] Ordonez G Rivas V Santos M Mondragon M Pineda B Rodriguez K. Herpes viruses in optic neuritis: similar to Bell's palsy. Clin Neurol Neurosurg. (2020) 188:105588. doi: 10.1016/j.clineuro.2019.105588, PMID: 31715425

[24] Phang DSK Ettikan JV Abd Aziz H Vendargon FM Sonny Teo KS. A rare complication of herpes zoster Ophthalmicus (HZO). Cureus. (2023) 15:e35693. doi: 10.7759/cureus.35693 37012964 PMC10066721

[25] Li R Zhong X Qiu W Wu A Dai Y Lu Z. Association between neuromyelitis optica and tuberculosis in a Chinese population. BMC Neurol. (2014b) 14:33. doi: 10.1186/1471-2377-14-33, PMID: 24555792 PMC3938476

[26] Beck RW Cleary PA. Optic neuritis treatment trial one-year follow-up results. Arch Ophthalmol. (1993) 111:773–5. doi: 10.1001/archopht.1993.01090060061023, PMID: 8512477

[27] Moss HE Gao W Balcer LJ Joslin CE. Association of race/ethnicity with visual outcomes following acute optic neuritis: an analysis of the optic neuritis treatment trial. JAMA Ophthalmol. (2014) 132:421–7. doi: 10.1001/jamaophthalmol.2013.7995, PMID: 24557028 PMC4115276

[28] Ishikawa HT Kezuka K Shikishima A Yamagami M Hiraoka H Chuman M. Epidemiologic and clinical characteristics of optic neuritis in Japan. Ophthalmology. (2019) 126:1385–98. doi: 10.1016/j.ophtha.2019.04.042, PMID: 31196727

[29] Kassan SS Moutsopoulos HM. Clinical manifestations and early diagnosis of Sjögren syndrome. Arch Intern Med. (2004) 164:1275–84. doi: 10.1001/archinte.164.12.1275 15226160

[30] Tang WQ Wei SH. Primary Sjögren's syndrome related optic neuritis. Int J Ophthalmol. (2013) 6:888–91. doi: 10.3980/j.issn.2222-3959.2013.06.26, PMID: 24392343 PMC3874534

[31] Nitescu D Nicolau A Caraiola S Predeteanu D Ionescu R Tanasescu C. Neuromyelitis optica--complication or comorbidity in primary Sjögren's syndrome? Rom J Intern Med. (2011) 49:295–300. PMID: 22568275

[32] Chou YS Lu DW Chen JT. Ankylosing spondylitis presented as unilateral optic neuritis in a young woman. Ocul Immunol Inflamm. (2011) 19:115–7. doi: 10.3109/09273948.2010.530732, PMID: 21428749

[33] Menon V Khokhar S. Ankylosing spondylitis in a case of recurrent optic neuritis. J Neuroophthalmol. (2001) 21:235. doi: 10.1097/00041327-200109000-00020, PMID: 11725198

[34] Zhao S Xu QG Zhu J Peng CX Li XM Zhou HF. Acute bilateral optic neuritis in active ankylosing spondylitis. Chin Med J. (2015) 128:2821–2. doi: 10.4103/0366-6999.167366, PMID: 26481754 PMC4736902

[35] Bando Y. Roads to formation of Normal myelin structure and pathological myelin structure. Adv Exp Med Biol. (2019) 1190:257–64. doi: 10.1007/978-981-32-9636-7_16, PMID: 31760649

[36] Gourraud PA Harbo HF Hauser SL Baranzini SE. The genetics of multiple sclerosis: an up-to-date review. Immunol Rev. (2012) 248:87–103. doi: 10.1111/j.1600-065X.2012.01134.x, PMID: 22725956 PMC5967887

[37] Handel AE Giovannoni G Ebers GC Ramagopalan SV. Environmental factors and their timing in adult-onset multiple sclerosis. Nat Rev Neurol. (2010) 6:156–66. doi: 10.1038/nrneurol.2010.1, PMID: 20157307

[38] Kefella H Luther D Hainline C. Ophthalmic and neuro-ophthalmic manifestations of sarcoidosis. Curr Opin Ophthalmol. (2017) 28:587–94. doi: 10.1097/ICU.0000000000000415, PMID: 28759560

[39] Yates WB McCluskey PJ Fraser CL. Neuro-ophthalmological manifestations of sarcoidosis. J Neuroimmunol. (2022) 367:577851. doi: 10.1016/j.jneuroim.2022.577851, PMID: 35405430

[40] Frohman LP Guirgis M Turbin RE Bielory L. Sarcoidosis of the anterior visual pathway: 24 new cases. J Neuroophthalmol. (2003) 23:190–7. doi: 10.1097/00041327-200309000-00002, PMID: 14504590

